# TMT-Based Proteomics Analysis Revealed the Protein Changes in Perirenal Fat from Obese Rabbits

**DOI:** 10.3390/ijms242417167

**Published:** 2023-12-06

**Authors:** Genglong Jiang, Jiahao Shao, Tao Tang, Meigui Wang, Jie Wang, Xianbo Jia, Songjia Lai

**Affiliations:** College of Animal Science and Technology, Sichuan Agricultural University, Chengdu 611130, China; jianggl1196282249@163.com (G.J.); shaojh1997@163.com (J.S.); m18483220592@163.com (T.T.); wmg1987797495@163.com (M.W.); wjie68@163.com (J.W.); jaxb369@sicau.edu.cn (X.J.)

**Keywords:** proteomics, high-fat diet, white adipose tissue, obesity

## Abstract

Obesity has become increasingly prevalent in recent years, and there is a need for a deeper understanding of the complex pathogenesis underlying the obesity condition. Therefore, the objective of this study was to investigate how a high-fat diet (HFD) affects protein expression in a female-rabbit model compared to a standard normal-diet group (SND), to gain comprehensive insights into the molecular mechanisms involved in obesity. To achieve this objective, a tandem mass tag (TMT)-based quantitative proteomics analysis was conducted to examine the molecular changes occurring in the white adipose tissue (WAT) from the HFD and SND groups. The sequencing results identified a total of 4215 proteins, among which 151 proteins exhibited significant differential expression. Specifically, there were 85 upregulated proteins and 66 downregulated proteins in the HFD group compared to the SND group. Further analysis of these differentially expressed proteins (DEPs) revealed their involvement in crucial biological processes, including energy metabolism, hormonal regulation, and inflammatory response. In conclusion, this study sheds light on the impact of HFD on protein expression in a female-rabbit model, providing new insights into the molecular mechanisms underlying obesity and the associated metabolic disorders.

## 1. Introduction

With society’s rapid development, dietary habits have undergone significant changes. Excessive consumption of a high-fat diet (HFD), including animal lard, cream, and fried foods, is a significant factor in the development of metabolic chronic diseases like obesity [1]. Obesity increases the risk of cardiovascular diseases and other types of cancer, posing a significant threat to individual health [2,3,4,5]. To mitigate these risks and prevent obesity, it is essential to embrace a healthy lifestyle [6,7]. However, over 90% of adult obese individuals are unaware of or do not receive proper management for their condition [8]. It is fascinating to note that research consistently demonstrates a higher prevalence of obesity among women compared to men in all age groups. These findings raise concerns about the future, as projections indicate that by 2025 the global obesity rate is expected to reach 18% among men and 21% among women [9]. Understanding the potential pathways that link obesity with abnormal gene expression related to metabolism is crucial, particularly for women. Gender differences in body composition are observed, with men having higher proportions of lean body mass and women having greater amounts of body fat. Men tend to accumulate fat around the trunk and abdomen, while women typically accumulate it around the hips and thighs [10,11]. Women’s heightened resistance to famine conditions also makes them more susceptible to being overweight, highlighting their vulnerability to the contemporary obesity epidemic [12]. Visceral adipose tissue is strongly associated with cardiometabolic risk factors in obese women, compared to other measures of body-fat distribution [13]. Inflammation in breast adipose tissue due to obesity may lead to altered estrogen signaling via increased expression and due to the activity of aromatase [14]. Obesity not only poses physical health risks for women but also has psychological consequences. Weight-related shame and teasing from peers, family members, and strangers can result in detrimental psychological, physical, and social consequences, thus further contributing to the increasing prevalence of obesity [15,16].

White adipose tissue (WAT) and brown adipose tissue (BAT) are the two main types of fat tissues in the body. BAT is characterized by multi-locular lipid droplets and abundant mitochondria, which give it the ability to generate heat through non-shivering thermogenesis [17]. Moreover, WAT can produce triglycerides for long-term storage and release free fatty acids from triglycerides when energy is needed [18]. However, when this balance is disrupted, excessive accumulation of body fat in WAT can lead to obesity. Dysfunction of WAT is associated with various metabolic disorders related to obesity, which can be characterized by impaired preadipocyte differentiation, increased oxidative stress and mitochondrial dysfunction, reduced vascularization and hypoxia, enhanced fibrosis, and accumulation of senescent cells [19]. WAT serves as a crucial source of leptin, a significant adipokine involved in obesity development [20]. Studies have demonstrated higher expression levels of leptin in WAT compared to BAT [21]. In murine models lacking leptin, notable observations include hyperphagia, obesity, and insulin resistance, highlighting the essential role of leptin in appetite regulation, energy balance, and insulin sensitivity [22,23]. Furthermore, research indicates that leptin not only plays a role in obesity management but also offers potential therapeutic applications. Leptin therapy has shown promise in treating and alleviating ectopic fat accumulation associated with obesity [24]. Moreover, due to its ability to inhibit insulin secretion, leptin could serve as an adjunctive treatment for individuals with insulin-deficient diabetes under specific conditions [25,26].

The expression level of proteins is most closely correlated with biological function phenotypic characteristics [27,28]. Friedrich and colleagues conducted TMT-based proteomic analysis on cancer samples, yielding significant insights into the variations, modifications, and interactions involved in cancer development and progression [29]. This provides valuable resources for a deeper understanding of the molecular mechanisms of cancer and personalized medicine. Based on the TMT method, researchers screened changes in serum protein profiles between patients with diabetic foot ulcers (DFU) and healthy controls and identified the activation of the extracellular matrix, complement system, and coagulation cascade as possible mechanisms implicated in the development of DFU [30]. Proteomics research plays a crucial role in revealing the development and progression of diseases, and it is particularly important in understanding the impact of obesity on women’s health. However, to our knowledge, only a limited number of studies have utilized obese female rabbits as a model to investigate the potential mechanisms underlying this impact. Therefore, this study aims to compare the differences in protein composition between two groups of rabbits: one group on a standard normal diet (SND) and the other group on a HFD group. By conducting an in-depth analysis of proteomic sequencing data from obese female rabbits, this study provides a broader perspective for obesity research, enhancing our understanding of the complexity of obesity and its associated metabolic disorders.

## 2. Results

### 2.1. Body-Weight and Biochemical-Indicator Disparity in the HFD and SND Groups

**Figure 1:**
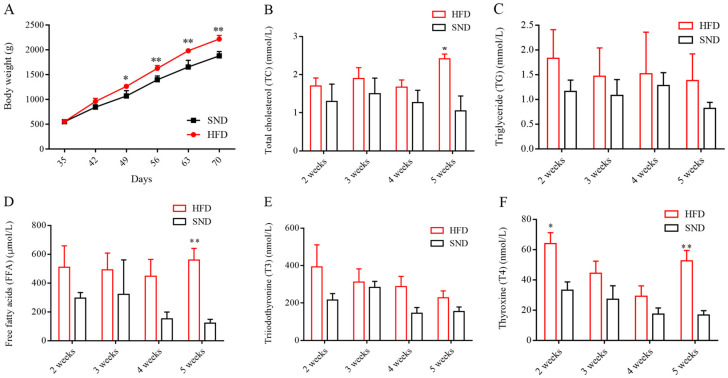
Body weight and serum indexes of the HFD and SND groups. (**A**) Changes in body weight of the HFD and SND groups over 5 weeks. Changes in total cholesterol (TC, (**B**)), triglycerides (TG, (**C**)), free fatty acids (FFA, (**D**)), triiodothyronine (T3, (**E**)), and thyroxine (T4, (**F**)) between the HFD and SND groups at weeks 5. n = 3 in (**A**–**F**). The data are presented as means ± SEM. * *p*-value < 0.05; ** *p*-value < 0.01.

At the outset of the test, there was no statistically significant difference in body weight between the HFD and SND groups (HFD: 551.7 ± 7.637 g; SND: 548.3 ± 7.637 g; Figure 1A). However, as the trial progressed, the disparity in weight between the two groups grew. The average body weight of HFD and SND rabbits exhibited significant divergence after 2 weeks (*p* < 0.05), and this discrepancy became highly significant at 3–5 weeks (*p* < 0.01). At the end of the experiment, we measured the serum indicators, and the results indicated higher levels of obesity-related markers such as TC, TG, FFA, and T4 in the HFD group compared to the SND group (Figure 1B–F). These findings collectively confirm the successful establishment of an obese rabbit model in this study.

### 2.2. Quality Control of TMT Proteomic Sequencing

**Figure 2:**
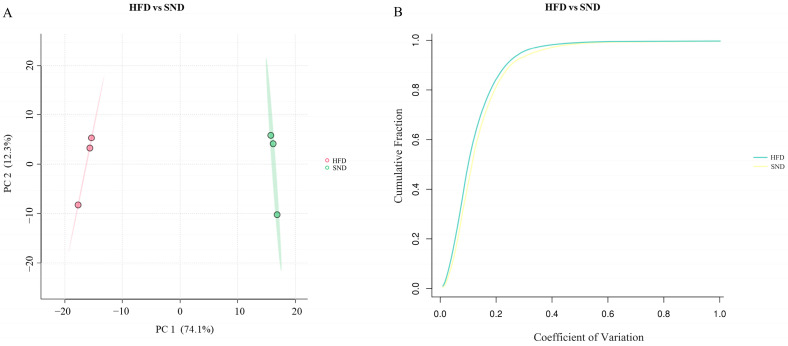
Quality control of TMT proteomic sequencing. (**A**) The *x*-axis PC1 and the *y*-axis PC2 represent the scores of the first and second principal components, respectively. The red color represents the HFD group, while the green color represents the SND group. (**B**) The cumulative plot of CV values for proteins in the HFD and SND groups. A steeper upward curve indicates better overall reproducibility of the samples.

We then collected WAT and performed proteomic sequencing analysis. The quality control results of the TMT protein mass spectrometry experiment indicate a comprehensive assessment of sample quality. Firstly, we performed Principal Component Analysis (PCA) to evaluate the differences between groups based on protein expression profiles. The PCA plot revealed distinct clustering among the groups, indicating significant differences in protein expression between groups (Figure 2A). Additionally, we calculated the Coefficient of Variation (CV) to assess technical reproducibility. The CV plot demonstrated low variation in protein expression within the groups, indicating that the samples have good technical reproducibility (Figure 2B).

### 2.3. Identification of Differentially Expressed Proteins (DEPs)

**Figure 3:**
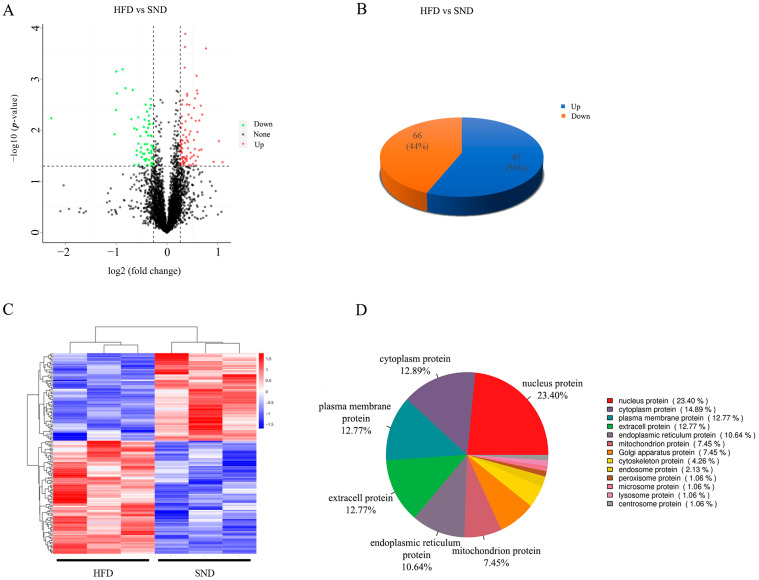
Identification of DEPs. (**A**) The volcano plot of identified proteins between the HFD and SND groups. (**B**) The distribution of upregulated and downregulated proteins in the DEPs that we obtained. (**C**) Hierarchical cluster analysis of DEPs between groups. (**D**) Subcellular localization analysis of DEPs.

By employing TMT protein quantification technology and tandem mass spectrometry analysis, Peptide Spectrum Matches (PSMs) with a credibility level above 99% were considered reliable PSMs. Proteins containing at least one unique peptide sequence were classified as credible proteins. Only the reliable PSMs and proteins were retained, and False Discovery Rate (FDR) validation was performed to eliminate peptides and proteins with an FDR greater than 1%. The SND group and HFD group yielded a total of 365,207 spectra, with 78,295 being matched. The total number of peptides was 38,912, while the total number of identified proteins was 5,589. Based on the credible proteins identified in the two sample groups, the selection of DEPs was performed based on the criteria (fold change (FC) ≥ 1.2 and *p* < 0.05 for up; FC ≤ 0.83 and *p* < 0.05 for down). A total of 4,215 proteins were identified, out of which there were 151 DEPs in the comparison group (Figure 3A, Supplementary Material S1). According to the criterion, 85 proteins (56%) were upregulated, and 66 proteins (44%) were downregulated (Figure 3B). DEPs with similar expression levels across different samples were screened and grouped using a hierarchical cluster analysis, which demonstrated good biological reproducibility between the two conditions (Figure 3C).

Subcellular localization refers to the specific location of a protein or its expression product within a cell. It is an important piece of information for studying protein function. Understanding the subcellular localization of proteins is crucial for understanding organisms. In this study, the subcellular localization analysis of DEPs was performed using the Cell-mPLOC 2.0 website. The results showed that nucleus proteins accounted for 23.40%, cytoplasm proteins accounted for 12.89%, plasma membrane proteins accounted for 12.77%, and extracell proteins accounted for 12.77% (Figure 3D).

### 2.4. Biological Information Analysis for DEPs

**Figure 4:**
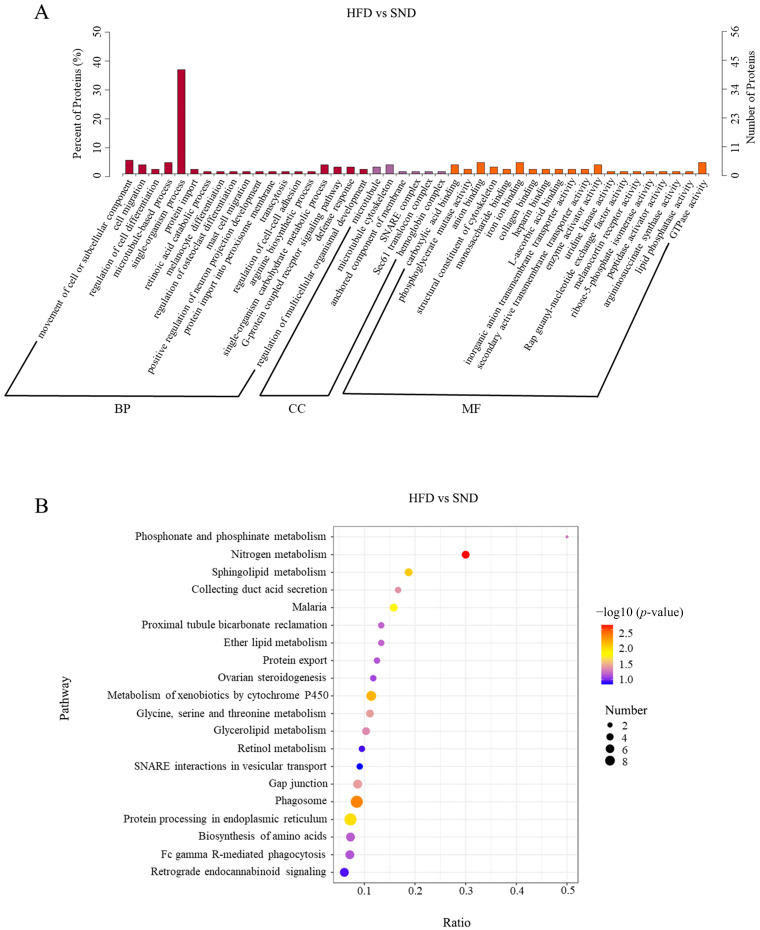
Biological information analysis for DEPs. (**A**) GO analysis was performed for the known DEPs, and the terms with significant enrichment in BP, CC, and MF categories were imaged. (**B**) The DEPs were enriched in the top 20 most significantly enriched KEGG pathways.

We performed a Gene Ontology (GO) functional annotation analysis on the obtained DEPs to further explore their biological functions. The results of the analysis for biological processes (BP), cellular components (CC), and molecular functions (MF) are shown in Supplementary Material S2. The analysis revealed 206 annotated GO terms, with 19 terms significantly enriched in BP, 5 terms significant enriched in CC, and 23 terms significant enriched in MF (Figure 4A). Among the significant GO terms, the categories with the highest number of DEPs included the single-organism process, movement of cell or subcellular component, microtubule-based process, microtubule, microtubule cytoskeleton, anchored component of membrane, anion binding, iron ion binding, and GTPase activity. To gain a deeper understanding of the metabolic and signaling pathways involved in the growth and development-related DEPs between the HFD and SND groups, we conducted a Kyoto Encyclopedia of Genes and Genomes (KEGG) pathway enrichment analysis on the DEPs (Supplementary Material S3). The top 20 pathways mapped with the DEPs are shown in Figure 4B. We found that 8 proteins (cytoskeleton associated protein 4, DnaJ heat shock protein family member C10, Oryctolagus cuniculus signal sequence receptor subunit 4 (SSR4), lectin mannose binding 1, SEC61 translocon alpha 1 subunit, ENSOCUP00000036470.1, ENSOCUP00000041431.1, ENSOCUP00000048909.1) were enriched in autophagy-related pathways, which may be associated with inflammation caused by obesity. Additionally, three proteins were found to be enriched in the Glycerolipid metabolism signaling pathway, including glycerate kinase, ENSOCUP00000034747.1, and ENSOCUP00000043755.1, all of which are related to lipid metabolism.

### 2.5. Network Analysis of PPI

**Figure 5:**
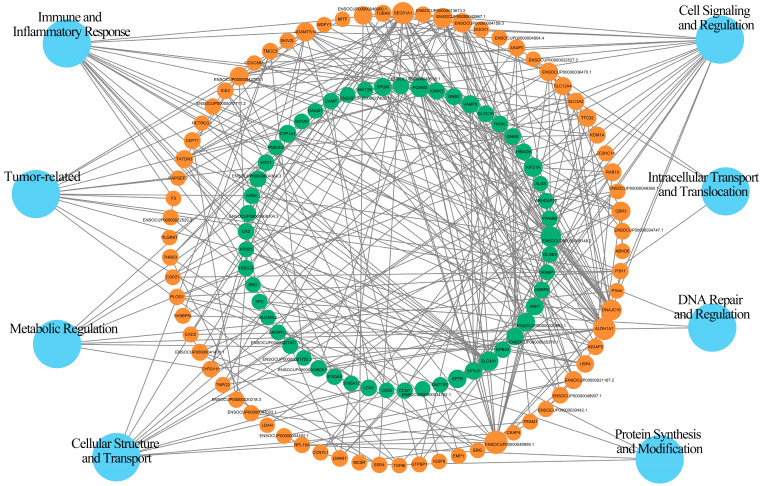
Network analysis of PPI. The size of the node represents the number of interacting proteins, with larger nodes indicating more interactions. The color of the node represents the expression level of the protein in the comparative analysis. Orange color indicates significantly high expression, while green color represents significantly low expression.

We constructed a PPI network for the DEPs using the STRING-db database (https://string-db.org/, accessed on 10 June 2020) and Cytoscape software (version 3.9.1, Figure 5). In this network, we utilized colors to represent the expression levels of the nodes. The nodes with significant upregulation are depicted in orange, while those with significant downregulation are shown in green. Among the DEPs, ENSOCUP00000048909.1 exhibited the highest degree of connectivity. Specifically, four proteins, namely melanocortin 5 receptor, DnaJ heat shock protein family (Hsp40) member C10, SEC61 translocon alpha 1 subunit, and ENSOCUP00000036470.1, showed the strongest interactions with ENSOCUP00000048909.1 in the network.

## 3. Discussion

The prevalence of being overweight and obesity has reached an epidemic level in Western countries [31], ranking as the second-leading preventable cause of death after tobacco use [32]. While there has been intense debate regarding the etiology of obesity, it is generally believed that weight gain and fat accumulation result from long-term energy intake exceeding energy expenditure [33]. However, this is not the sole determining factor, as obesity depends on genetic factors inherited from parents [34]. Although genetic factors undeniably play a role in individual weight gain and obesity, it has been established that many genetic loci associated with obesity risk only account for a small fraction of the causative factors behind obesity [35]. Numerous genetic factors remain unexplained, which has sparked growing interest in understanding the potential role of genetic factors in contributing to obesity. Research indicates that obesity significantly induces the production of pro-inflammatory adipokines, while adipose tissue secretes corresponding anti-inflammatory cytokines (such as IL-4, IL-10, IL-13, IL-19) [36]. These anti-inflammatory cytokines decrease with increasing body weight, thus making adipose tissue the primary source of inflammatory mediators [2,37]. Furthermore, a significant number of patients with unexplained leukocytosis, a hallmark of inflammation, show no identifiable causes apart from obesity. Interestingly, leukocytosis appears to be more predominant in females compared to males [38]. Therefore, unraveling this scenario is crucial for understanding women’s health. To delve further into the mechanisms underlying the impact of genetic factors on obesity development, we established an obesity model by feeding female rabbits a HFD. Using TMT proteomics sequencing technology, we analyzed the differences in protein composition between the HFD and the SND groups, specifically focusing on WAT. This approach allows us to understand some molecular-level changes in the development of obesity by investigating the protein profiles derived from WAT.

In this study, a total of 4215 proteins were identified, including 151 DEPs (85 upregulated proteins and 66 downregulated proteins). In all the obtained upregulated and downregulated proteins, Aldehyde dehydrogenase 1 family member A1 (ALDH1A1), which is responsible for converting retinol into retinoic acid, is a significant therapeutic target for treating debilitating disorders like cancer, obesity, and inflammation [39,40]. It is associated with cellular oxidative stress and antioxidant responses. It is considered a tumor-initiating cell marker in many cancers, including breast cancer. Research has shown that the induction of ALDH1A1 enzyme activity can promote the expansion of myeloid-derived suppressor cells and plays a role in triggering the pre-cancerous immune microenvironment, promoting breast cancer progression, and ultimately leading to tumor growth. It is also associated with neurodegenerative diseases and immune regulation [41,42]. The upregulation of ALDH1A1 in the HFD group indicates changes in lipid metabolism and inflammatory status within the body. It also suggests an increased risk of obesity-related cancer. On the other hand, S100 calcium binding protein A12 (S100A12) is a biomarker that has been proven useful during inflammatory conditions, and it is also significantly associated with obesity and metabolic syndrome [43,44]. S100A12 is a biomarker that has been shown to be useful under inflammatory conditions. Decreased levels of S100A12 may be related to abnormal immune system function and the development of chronic inflammation. Differential protein lipocalin 2 (LCN2) is a member of the adipokine protein family and is associated with various diseases, including cancer, diabetes, and obesity [45,46]. Functionally, LCN2 is considered an anti-inflammatory protein as the LCN2 gene’s promoter region contains nuclear factor-κB (NF-κB) binding sites [47,48]. Therefore, the downregulation of LCN2 may indicate an increase in inflammatory response in the HFD group. IQGAP2 is upregulated as a differentially expressed protein and has been shown to be associated with pro-inflammatory responses [49]. On the other hand, in terms of its role in cancer, several studies have suggested that IQGAP2 may act as a tumor-suppressor gene [50]. In addition to the differential proteins mentioned above, there are several other proteins that are associated with inflammatory responses. These include S100 calcium binding protein A8 (S100A8), Rho GTPase activating protein 25 (ARHGAP25), Endoglin (ENG) and CD93 molecule (CD93) [51,52,53,54]. These proteins may play important roles in regulating cytokine production, inflammatory signaling pathways, and other relevant biological processes during the regulation of inflammation. In addition to ALDH1A1, there are several other differential proteins that may be associated with cancer development. These include carbonyl reductase 3, epithelial membrane protein 1, solute carrier family 3 member 2, Ankyrin 1, Mannose binding lectin 2, cancer susceptibility 4, Deoxynucleotidyltransferase terminal interacting protein 2, macrophage stimulating 1, rho GTPase activating protein 25, and coiled-coil domain containing 88A [55,56,57,58,59,60,61,62]. These proteins are likely involved in the regulation of various aspects of cancer, and they may have a significant impact on the occurrence and development of cancer.

The analysis by GO indicates that DEPs in the obesity model induced by a HFD are enriched in various biological processes and molecular mechanisms, such as movement of the cell or subcellular component, microtubule-based process, anchored component of membrane, and metabolic regulation. This suggests a close relationship between these biological processes, molecular mechanisms, and the development of obesity. It provides important clues for further investigation into the molecular mechanisms of obesity and potential therapeutic targets. In our study, we observed two pathways, glycerolipid metabolism, and ether lipid metabolism pathway, through KEGG pathway enrichment analysis. These pathways are believed to have significant involvement in the lipid metabolism and cell differentiation processes [63,64,65]. The glycerolipid metabolism pathway is highly associated with several cancer hallmark pathways and immune microenvironments as well [66]. This suggests that these pathways may play a role in tumor development. Further research will help reveal the potential therapeutic and immunotherapeutic applications of these pathways in cancer treatment. Additionally, the phagosome pathway is related to immune and inflammatory responses [67]. When the body is damaged, macrophages gather at the site of injury, extend pseudopods, and form invaginated vesicles called phagosomes through endocytosis [68]. Phagosomes have the ability to engulf exogenous toxic substances and generate a substantial amount of inflammatory factors and chemotactic factors to respond to and defend against potential damage [69]. As mentioned earlier, the enrichment of DEPs in pathways may be associated with the consideration of obesity as a chronic inflammatory condition [70]. In addition, it has been observed in obese individuals that obesity induced by a HFD triggers an increase in the production of chemokine (C-C motif) ligand 2/monocyte chemoattractant protein-1 (CCL2/MCP-1) within epithelial cells, ultimately leading to the development of a proinflammatory phenotype [71,72].

The Sphingolipid metabolism pathway regulates various cellular biological processes including growth regulation, cell migration, adhesion, apoptosis, aging, and inflammatory response. It also plays a role in metabolic disorders, various cancers (and their attributes), immune function, and cardiovascular diseases [73,74,75]. Disruption of the sphingolipid metabolism may lead to abnormal function of adipocytes. The metabolism of xenobiotics by the cytochrome P450 pathway plays an important role in drug detoxification, cellular metabolism, and homeostasis [76]. Obese patients often require medication, and the obese state may affect the expression and activity of these enzymes, thereby altering the metabolism rate and efficacy of drugs.

The results obtained in a small population of animals serve as an initial exploration into the association between proteomic remodeling and diet-induced effects. However, it is important to recognize that these findings should be interpreted with caution due to several limitations inherent in our study design. Firstly, the small sample size used in our study may limit the generalizability of our results to larger populations. The variability within a small sample may lead to biased or less-robust conclusions. Therefore, future studies with larger and more diverse animal cohorts are warranted to validate our findings and provide a more comprehensive understanding of the relationship between proteomic remodeling and diet-induced effects. Secondly, while we have discussed potential issues related to proteomic remodeling and diet-induced effects, it is crucial to acknowledge that our findings are based on associative observations. Without mechanistic inferences, we cannot establish a direct cause-and-effect relationship. To overcome this limitation, future research should incorporate more in-depth mechanistic investigations, such as functional assays or pathway analyses, to elucidate the underlying biological mechanisms. Furthermore, it is worth noting that our study focused solely on animal models, and translation to human subjects should be approached cautiously. Species differences and variations in metabolism may affect the applicability of our findings to human populations. Future studies involving human subjects or in vitro experimentation could provide valuable insights into the relevance of our findings in the context of human health. In conclusion, the limitations inherent in our study, including the small sample size, lack of mechanistic inferences, and the need for further validation in diverse populations, must be recognized. Despite these limitations, our study offers valuable preliminary insights into the association between proteomic remodeling and diet-induced effects. It sets the stage for future research that may address these limitations and provide a more comprehensive understanding of this complex relationship.

## 4. Materials and Methods

### 4.1. Animals

**Figure 6:**
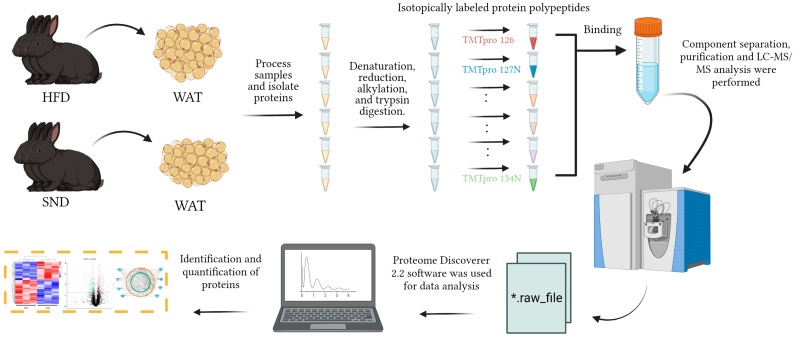
The diagram provides a detailed overview of the experiment, highlighting the steps involved in the study, from the underlying principle to the establishment of the sample model, followed by data acquisition and subsequent data analysis.

All experiments in the present study involving animals were performed under the direction of the Institutional Animal Care and Use Committee from the College of Animal Science and Technology, Sichuan Agricultural University, China (Certification No. SYXK2019-187). We use the obese-rabbit model was established using HFD. Briefly, 6 female Tianfu black rabbits from a breeding strain maintained by Sichuan Agricultural University were 35 days old at the initiation of the study. The animals were randomly divided into two groups and fed a SND or a HFD for 5 weeks. The nutritional breakdown of the SND and HFD groups was described in Supplementary Material S4. Each rabbit was kept separately in a clean cage (600 × 600 × 500 mm) and placed in an environmentally controlled room (21–23 °C, 60–75% humidity, 14 h light [60 lx]). Water was freely available and feed (120 g/d) was provided twice a day. All animals were raised at the teaching farm of Sichuan Agricultural University. The experimental strategy is shown in Figure 6.

### 4.2. Measuring Blood Markers

Blood samples were collected from overnight-fasted animals the following morning via the auricular veins using vacutainer tubes. After collection, the samples were immediately centrifuged at 4 °C for 5 min to separate the serum. The resulting serum was carefully transferred to clean frozen tubes and stored at −80 °C until further analysis. The levels of TC, TG, FFA, and T4 in the serum were determined using specific assay kits. The TC, TG, and T4 assay kits were purchased from Nanjing Jiancheng Bioengineering Institute (Nanjing, China), while the FFA assay kit was provided by Solarbio Science & Technology Co., Ltd. (Beijing, China). The measurements were performed according to the manufacturer’s instructions.

### 4.3. Total Protein Extraction

Adipose tissue samples taken from the perirenal area of rabbits were ground individually in liquid nitrogen and lysed with lysis buffer containing 100 mM NH_4_HCO_3_ (pH = 8), 8 M Urea, and 0.2% SDS, and then sonicated on ice for 5 min. The lysate was centrifuged at 12,000× *g* for 15 min at 4 °C, and the supernatant was transferred to a clean tube. Extracts from each sample were reduced with 10 mM DTT for 1 h at 56 °C and subsequently alkylated with sufficient iodoacetamide for 1 h at room temperature in the dark. Then, samples were completely mixed 4 times with a volume of precooled acetone by vortexing and were incubated at −20 °C for at least 2 h. Samples were then centrifuged, and the precipitation was collected. After washing twice with cold acetone, the pellet was dissolved using dissolution buffer, which contained 0.1 M triethylammonium bicarbonate (TEAB, pH = 8.5) and 6 M urea.

### 4.4. TMT Labeling of Peptides

Each protein sample was taken at a quantity of 120 μg, and the volume was adjusted to 100 μL using dissolution buffer. To the sample, 1.5 μg of trypsin and 500 μL of 100 mM TEAB buffer were added. The mixture was then mixed and digested at 37 °C for 4 h. Then, an additional 1.5 μg of trypsin and CaCl_2_ were added to the sample. The sample was left to digest overnight. Formic acid was mixed with the digested sample, adjusted to a pH under 3, and centrifuged at 12,000× *g* for 5 min at room temperature. The supernatant was slowly loaded to the C18 desalting column, washed with washing buffer (0.1% formic acid, 3% acetonitrile) 3 times, and then eluted with some elution buffer (0.1% formic acid, 70% acetonitrile). The eluents of each sample were collected and lyophilized. To reconstitute the sample, 100 μL of 0.1 M TEAB buffer was added. Afterward, 41 μL of the TMT labeling reagent dissolved in acetonitrile was added. The sample was then mixed by shaking at room temperature for 2 h. Then, the reaction was stopped by adding 8% ammonia. All labeling samples were mixed with equal volume, desalted, and lyophilized.

### 4.5. Separation of Fractions

Mobile phase A (2% acetonitrile, adjusted pH to 10.0 using ammonium hydroxide) and B (98% acetonitrile) were used to develop a gradient elution. The lyophilized powder was dissolved in solution A and centrifuged at 12,000× *g* for 10 min at room temperature. The sample was prepared using a C18 column (Waters BEH C18, 4.6 × 250 mm, 5 μm) on the Rigol L 3000 HPLC system. The column temperature was set to 50 °C. The eluates were monitored at UV 214 nm, collected for a tube per minute and combined into 10 fractions, finally. All fractions were dried under a vacuum, and then reconstituted in 0.1% (*v*/*v*) formic acid in water.

### 4.6. LC-MS/MS Analysis

For transition library construction, shotgun proteomics analyses were performed using an EASY-nLCTM 1200 UHPLC system (Thermo Fisher Scientific, Waltham, MA, USA) coupled with a Q Exactive HF-X mass spectrometer (Thermo Fisher Scientific, Waltham, MA, USA) operating in the data-dependent acquisition (DDA) mode. 1 μg sample was injected into a C18 nano-trap column (2 cm × 75 μm, 3 μm). Peptides were separated in an analytical column (15 cm × 150 μm, 1.9 μm). The separated peptides were analyzed using a Q Exactive HF-X mass spectrometer (Thermo Fisher Scientific, Waltham, MA, USA), with an ion source of Nanospray Flex™ (ESI), spray voltage of 2.3 kV, and ion transport capillary temperature of 320 °C. With a full scan range from *m*/*z* 350 to 1500 with resolution of 60,000 (at *m*/*z* 200), the automatic gain control (AGC) target value was 3 × 10^6^ and the maximum ion injection time was 20 ms. The top 40 precursors of the highest abundance in the full scan were selected and fragmented using higher energy collisional dissociation and analyzed in MS/MS, where resolution was 45,000 (at *m*/*z* 200) for 10 plex; the AGC target value was 5 × 10^4^; the maximum ion injection time was 86 ms; a normalized collision energy was set as 32%; an intensity threshold was 1.2 × 10^5^; and the dynamic exclusion parameter was 20 s.

### 4.7. The Identification and Quantitation of Protein

The Proteome Discoverer version 2.2 (PD; Thermo Fisher Scientific, Waltham, MA, USA) was utilized to acquire the MS/MS results. The search parameters were set as follows: a mass tolerance of 10 ppm for the precursor ion and a mass tolerance of 0.02 Da for the production. Carbamidomethyl was defined as a fixed modification, while methionine oxidation and TMT plex were specified as dynamic modifications. Acetylation and TMT plex were designated as N-terminal modifications in PD 2.2. A maximum of 2 miscleavage sites were allowed. To improve the quality of analysis results, the software PD 2.2 further filtered the retrieval results: PSMs with credibility of more than 99% were identified PSMs. The identified protein contains at least 1 unique peptide. The identified PSMs and protein were retained and performed with a FDR of no more than 1.0%. The protein quantitation results were statistically analyzed using *t*-test. The proteins with FC ≥ 1.2 or FC ≤ 0.83 and *p* < 0.05 were defined as DEPs.

### 4.8. The Functional Analysis of DEPs

The DEPs were subjected to functional analysis using the GO database through the interproscan program [77]. Wolf Psort (https://wolfpsort.hgc.jp/, accessed on 10 June 2020) was used to predict subcellular localization for proteins. Additionally, protein families and pathways were analyzed using the KEGG database [78]. The PPI were predicted using the STRING-db server (http://string.embl.de/, accessed on 10 June 2020) [79].

## 5. Conclusions

In summary, through TMT proteomic sequencing analysis of an HFD-induced obese-female-rabbit model, we identified DEPs including ALDH1A1, S100A12, LCN2, IQGAP2, S100A8, ARHGAP25, ENG, CD93, and other proteins. These proteins are associated with lipid metabolism, inflammatory response, and cancer development, and likely play crucial roles in the development of obesity. Additionally, enrichment analysis revealed that glycerolipid metabolism, ether lipid metabolism, and phagosome pathways are related to obesity. These findings provide potential therapeutic targets for further investigation into the molecular mechanisms of obesity and the development of strategies for the treatment and prevention of female obesity.

## Data Availability

All data generated or analyzed during this study are included in this published article.

## Supplementary Materials

The following supporting information can be downloaded at https://www.mdpi.com/article/10.3390/ijms242417167/s1.
