# Supplementary material for: TMT-Based Proteomics Analysis Revealed the Protein Changes in Perirenal Fat from Obese Rabbits
Source: Int J Mol Sci. 2023 Dec 6;24(24):17167. doi: 10.3390/ijms242417167 (PMC10743514; doi:10.3390/ijms242417167)
Supplement: Supplementary file 1 [file ijms-24-17167-s001.zip › Supplementary material S4.pdf]

Table S1. Composition and nutrient content of the standard normal diet (SND) and the high-fat diet (HFD)

Ingredient	SND							HFD						
	Proportion(%)	DE(MJ/kg)	CP(%)	EE(%)	CF(%)	Ca(%)	P(%)	Proportion(%)	DE(MJ/kg)	CP(%)	EE(%)	CF(%)	Ca(%)	P(%)
Straw powder	26	0.855	1.248	0.364	7.748	0.073	0.021	23.5	0.773	1.128	0.329	7.000	0.066	0.019
Maize	18	2.889	1.602	0.648	0.576	0.005	0.070	16	2.568	1.424	0.576	0.512	0.004	0.062
Barley	20	2.808	2.040	0.34	0.860	0.020	0.093	18	2.527	1.836	0.306	0.774	0.018	0.084
Bran	15	1.631	2.310	2.475	0.765	0.050	0.072	13.5	1.468	2.079	2.228	0.689	0.045	0.065
Bean cake	16	2.166	6.768	0.304	0.576	0.045	0.091	14.5	1.963	6.134	0.276	0.522	0.041	0.082
Fish meal	3.5	0.552	2.047	0.196		0.137	0.104	3.15	0.497	1.842	0.176		0.123	0.094
Lard								10	3.683		9.8			
Stone powder	1.0					0.350		0.9					0.315	
Salt	0.5							0.45						
Total	100	10.91	16.015	4.327	10.525	0.68	0.431	100	13.479	14.443	13.691	9.497	0.621	0.406

Note. DE: digestible energy; MJ: megajoule; CP: crude protein; EE: ether extract; CF: crude fiber; Ca: calcium; P: phosphorus.
